# Trends in Alcohol-Induced Deaths in the United States, 2000-2016

**DOI:** 10.1001/jamanetworkopen.2019.21451

**Published:** 2020-02-21

**Authors:** Susan Spillane, Meredith S. Shiels, Ana F. Best, Emily A. Haozous, Diana R. Withrow, Yingxi Chen, Amy Berrington de González, Neal D. Freedman

**Affiliations:** 1 Metabolic Epidemiology Branch, Division of Cancer Epidemiology and Genetics, National Cancer Institute, National Institutes of Health, Bethesda, Maryland; 2 Division of Cancer Treatment and Diagnosis, National Cancer Institute, National Institutes of Health, Bethesda, Maryland; 3 Pacific Institute for Research and Evaluation, Calverton, Maryland

## Abstract

**Importance:**

Notable increases in mortality from alcohol-induced causes over the past 2 decades in the United States have been reported. However, comprehensive assessments of trends in alcohol-induced mortality by sex, age, race/ethnicity, and social and geographic factors are lacking.

**Objective:**

To examine trends in alcohol-induced mortality rates from 2000 to 2016, comparing results by demographic characteristics including sex, race/ethnicity, age, county-level socioeconomic status, and geographic location.

**Design, Setting, and Participants:**

This serial cross-sectional study used US national vital statistics data for years 2000 to 2016 for all US residents older than 15 years. Data analysis was conducted from January to September 2019.

**Exposures:**

Trends in alcohol-induced mortality by sex, race/ethnicity, age, county-level socioeconomic status (ie, median income, percentage of unemployed residents, percentage of residents with a bachelor’s degree), rurality level, and US state.

**Main Outcomes and Measures:**

Alcohol-induced mortality, ie, deaths for which alcohol holds a population-attributable fraction of 1. Deaths were expressed per 100 000 residents as absolute and age-standardized rates. Mortality trends were measured as average annual percentage changes (AAPCs) for the entire period (ie, 2000-2016) and annual percentage changes (APCs) for individual periods of change within the study period.

**Results:**

A total of 425 045 alcohol-induced deaths were identified from 2000 to 2016 (2000: 19 627 deaths; 14 979 [76.3%] men; 2016: 34 857 deaths; 25 213 [73.3%] men). The rate of alcohol-induced deaths increased substantially among men (AAPC, 1.4%; 95% CI, 1.0% to 1.8%) and women (AAPC, 3.1%; 95% CI, 2.6% to 3.6%) and accelerated recently (men, 2012-2016: APC, 4.2%; 95% CI, 3.1% to 5.3%; women, 2013-2016: APC, 7.1%; 95% CI, 5.1% to 9.1%). The largest increases by race/ethnicity were observed among American Indian and Alaska Native men (AAPC, 3.3%; 95% CI, 2.6% to 4.0%), American Indian and Alaska Native women (AAPC, 4.2%; 95% CI, 3.8% to 4.6%), and white women (AAPC, 4.1%; 95% CI, 3.6% to 4.7%). Despite initial declines among black women, black men, and Latino men (eg, Latino men, 2000-2003: APC, −5.1%; 95% CI, −9.8% to −0.1%; 2003-2013: APC, −0.6%; 95% CI, −1.4% to 0.2%), increases occurred later in the study period (eg, Latino men, 2013-2016: APC, 4.1%; 95% CI, 0.3% to 8.1%). The rates of increase varied by age group and in turn by racial/ethnic group. Among white individuals, large absolute increases occurred in midlife (eg, men aged 55-59 years, 2000-2003: 25.5 deaths per 100 000 residents; 2013-2016: 43.3 deaths per 100 000 residents; women aged 50-54 years, 2000-2003: 7.4 deaths per 100 000 residents; 2013-2016: 16.5 deaths per 100 000 residents), although APCs were also large for ages 25 to 34 years, ranging from 4.6% to 6.9% per year among men and from 7.3% to 12.0% among women. Among American Indian and Alaska Native individuals, increases throughout the age range were observed, with the largest absolute increase occurring for ages 45 to 49 years among men (2000-2013: 113.6 deaths per 100 000 residents; 2013-2016: 193.1 deaths per 100 000 residents) and for ages 50 to 54 among women (2000-2013: from 56.1 deaths per 100 000 residents; 2013-2016: 105.1 deaths per 100 000 residents).

**Conclusions and Relevance:**

This study found large increases in alcohol-induced death rates across age and racial/ethnic subgroups of the US population, which have accelerated over recent years. Large increases in alcohol-induced deaths among younger age groups may be associated with future increases in alcohol-related disease.

## Key Points

**Question:**

How have rates of alcohol-induced deaths changed in recent years among different US population subgroups?

**Findings:**

In this serial cross-sectional study using US mortality data from 2000 to 2016, large increases in alcohol-induced mortality among both men and women were observed. Despite initial decreases among some groups, significant increases in mortality were observed among all racial/ethnic groups in the years 2013 to 2016.

**Meaning:**

The recent acceleration of alcohol-induced deaths observed in this study indicates a broad public health challenge worthy of urgent attention.

## Teaser

This serial cross-sectional study examines trends in alcohol-induced mortality rates in the United States from 2000 to 2016, comparing results by demographic characteristics including sex, race/ethnicity, age, county-level socioeconomic status, and geographic location.

## Introduction

Increases in mortality from alcohol-induced causes have been reported in the United States during the past 2 decades.^1^ Recent reports have largely focused on the contributions of alcohol to increasing rates of premature mortality among white US residents aged 25 to 64 years.^2,3,4,5^ However, increases have also been reported among other groups.^1,3,5^ Comprehensive assessments of trends in alcohol-induced mortality by sex, age, and race/ethnicity are lacking.

Alcohol-induced deaths include the subset of alcohol-related deaths that are certain to be caused by drinking alcohol and therefore serve as indicators of the far larger spectrum of deaths, including traffic collisions and cancer, that often cannot be clearly classified as being caused by alcohol. Rates of alcohol-induced deaths are consequently markers of a far larger public health problem.

Therefore, monitoring rates of alcohol-induced deaths over time within sociodemographic subgroups of the US population is critical for targeting preventive health and health care resources.^6^ In this serial cross-sectional study, we analyzed patterns of alcohol-induced mortality within the United States between 2000 and 2016. We examined trends in alcohol-induced death rates over time, comparing results by demographic characteristics including sex, race/ethnicity, age, county-level socioeconomic status (SES), and geographic location.

## Methods

### Data Source and Study Population

This study used mortality data and associated demographic characteristics for the entire US population, derived from death certificates from the US National Center for Health Statistics, Centers for Disease Control and Prevention, for the years 2000 to 2016, inclusive. Population data were obtained from the US Census Bureau. For this study, we included members of the full cohort of the US population older than 15 years, the age cutoff commonly used by the World Health Organization and the Centers for Disease Control and Prevention in reports on alcohol consumption and health effects.^7,8^ We restricted analyses of the American Indian and Alaska Native (AIAN) population to Indian Health Service Purchased and Referred Care Service Delivery Area counties to minimize misclassification within this population.^9^ As the data were publicly available, institutional review board approval and informed consent were not needed, per the institutional policy of the National Cancer Institute. Reporting of this study followed the Strengthening the Reporting of Observational Studies in Epidemiology (STROBE) reporting guideline.

### Variables

Alcohol-induced deaths were defined conservatively as deaths for which alcohol held a population attributable fraction of 1, ie, deaths that are, by definition, due to alcohol consumption and could be avoided if alcohol were not involved.^10^ Such deaths were identified from the underlying cause of death recorded in death certificates using the 14 following *International Statistical Classification of Diseases and Related Health Problems, Tenth Revision *(*ICD*-*10*) codes, as used by the Centers for Disease Control and Prevention to quantify alcohol-induced deaths^1^: alcohol-induced pseudo-Cushing syndrome (E24.4); mental and behavioral disorders due to alcohol use (F10); degeneration of nervous system due to alcohol (G31.2); alcoholic polyneuropathy (G62.1); alcoholic myopathy (G72.1); alcoholic cardiomyopathy (I42.6); alcoholic gastritis (K29.2); alcoholic liver disease (K70); alcohol-induced acute pancreatitis (K85.2); alcohol-induced chronic pancreatitis (K86.0); finding of alcohol in blood (R78.0); accidental poisoning by and exposure to alcohol (X45); intentional self-poisoning by and exposure to alcohol (X65); and poisoning by and exposure to alcohol, undetermined (Y15).

Sex, age, and race/ethnicity were ascertained from death certificates as originally recorded by funeral directors. In line with the US Census, we categorized race/ethnicity as follows: non-Latino white, non-Latino black, Latino, Asian and Pacific Islander (API), and AIAN.^11^ Death certificate and census data collect ethnicity information using the category Spanish/Hispanic/Latino; we used the term *Latino* for this category. County-level SES was examined using data from the American Community Survey (2011-2015). These variables included unemployment percentage (ie, percentage of civilians aged ≥16 years in the labor force who are unemployed), percentage of population aged 25 years or older with a bachelor’s degree, and median household income in the past 12 months, measured in 2015 inflation-adjusted dollars. Variables were classified into quintiles according to their distribution across counties, by county population size, as described previously.^12^ We also examined rurality by categorizing counties using the 2013 Rural-Urban Continuum codes developed by the US Department of Agriculture.^13^

### Statistical Analysis

Mortality rates for each year of the study period were calculated using SEER*Stat version 8.3.5 (National Cancer Institute), standardized to the 2000 US standard population in 5-year age categories. Rates were calculated separately for men and women for all alcohol-induced deaths, individual causes of alcohol-induced death, and within race/ethnicity categories. The Joinpoint Regression Program version 4.6.0 (National Cancer Institute)^14^ was used to quantify the overall trends from 2000 to 2016 expressed as average annual percentage changes^15^ (AAPCs), to detect statistically significant changes in the trajectory of death rates, and to quantify the trends in these segments (expressed as annual percentage changes [APCs]). Statistically significant changes in trend were identified using the Monte Carlo permutation method at a threshold of *P* < .05.^16^ We examined potential differences by age in 2 ways. First, age-specific rates, based on 5-year age groups, were calculated for each of 2 periods (ie, 2000-2003 and 2013-2016). These 4-year periods allowed for comparison of age-specific rates between the most recent vs the oldest years within the study period while avoiding instability of data from potentially outlying individual years. Second, we used the age-period-cohort analysis webtool developed by Rosenberg et al^17^ to identify APCs in mortality rates during the study period for individual ages. Age-period-cohort models separate trends into age associations, which reflect natural history; period associations, which may reflect changes in the environment that affect all age groups; and cohort associations, which reflect differences in risk across birth cohorts. The age-period-cohort analysis webtool implements a panel of estimable age-period-cohort functions and corresponding Wald tests in R code following the input of age-specific numbers of events and person-years over time, which were uploaded to the tool in a comma-separated values format. For this analysis, we limited the cohort to the population aged 20 to 80 years because of low case numbers and statistical instability among age groups outside this range.

Additional independent variables under investigation included county-level SES and US state. Because of low numbers of deaths in some racial/ethnic categories, county-level analyses were limited to white, black, and Latino individuals, and state-level analyses were limited to white individuals. For analysis by county-level SES, AAPCs were calculated by individual level of each of county-level attribute (ie, median income, unemployment percentage, percentage of residents with a bachelor’s degree, rurality indicator). For analysis by US state, age-standardized rates for men and women were calculated for each state for 2000 to 2003 and 2013 to 2016. Rates were depicted using choropleth maps with classification by quantiles (n = 8). Additionally, the rate ratio, 2013 to 2016 vs 2000 to 2003, was calculated for each state.

## Results

This study used national mortality and population data; therefore, it included the full US population, which increased from 221.9 million individuals in 2000 to 262.2 million individuals in 2016. The distribution of men and women was relatively stable during this time (2000: 51.5% women; 2016: 51.2% women), whereas the distribution by race/ethnicity changed somewhat (2000: 72.2% white; 11.3% Latino; 11.6% black; 4.1% API; and 0.5% AIAN; 2016: 64.5% white; 16.0% Latino; 12.6% black; 6.1% API; and 0.5% AIAN).

**Figure 1.**
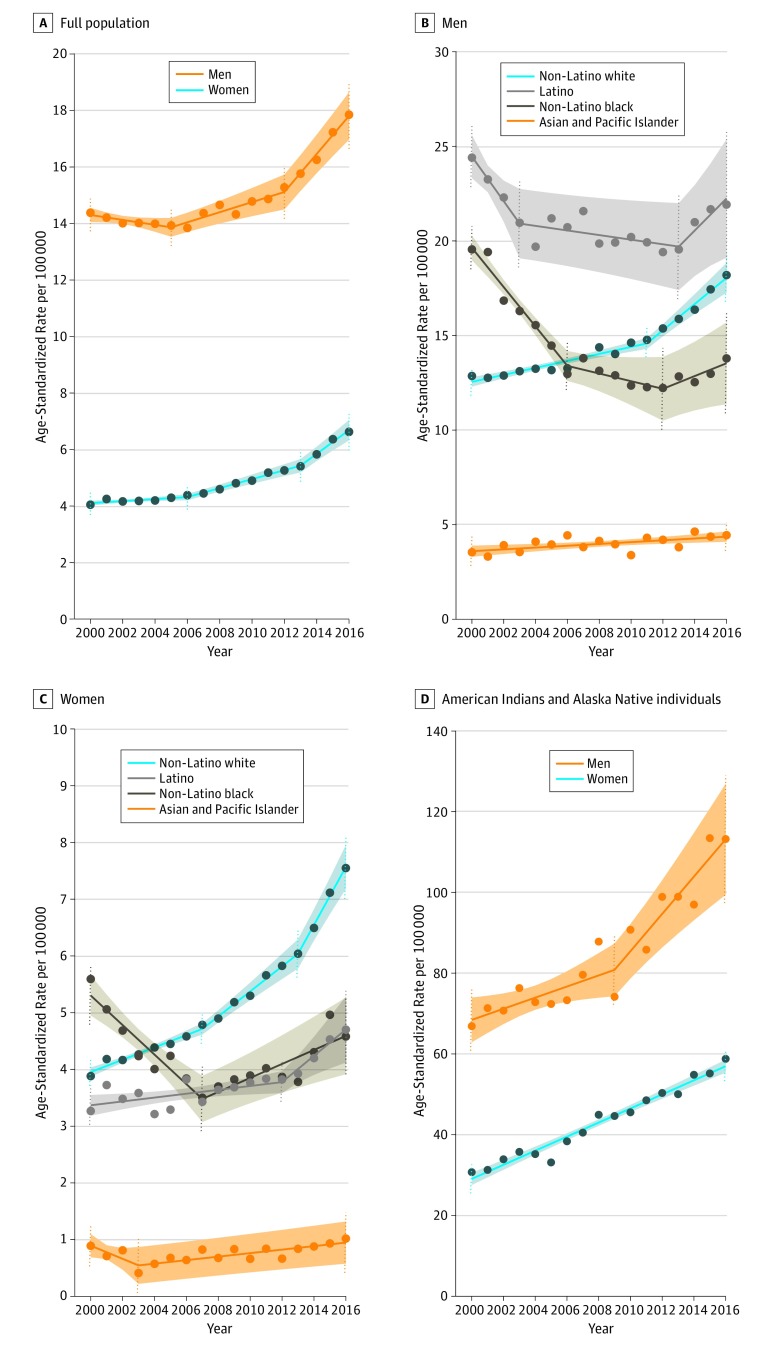
Age-Standardized Rates of Alcohol-Induced Death, 2000-2016 Observed age-standardized rates for each year are plotted using dotted markers. Solid lines are plotted from joinpoint analysis and indicate periods of change, with shaded areas indicating 95% CIs. Dotted vertical lines indicate time interval boundaries.

A total of 425 045 alcohol-induced deaths were identified from 2000 to 2016. In 2000, there were 19 627 deaths (14 979 [76.3%] men; 4649 [23.7%] women) in the US population from alcohol-induced causes, occurring at an age-standardized rate of 8.9 deaths per 100 000 residents (men: 14.4 deaths per 100 000 residents; women: 4.0 deaths per 100 000 residents) (Figure 1). By 2016, there were 34 857 deaths (25 213 [72.3%] men; 9644 [27.7%] women) with alcohol-induced causes at an age-standardized rate of 12.0 deaths per 100 000 residents (men: 17.9 deaths per 100 000 residents; women: 6.6 deaths per 100 000 residents). The age-standardized rates for each year from 2000 to 2016 by individually contributing cause of alcohol-induced death are presented in eFigure 1 in the Supplement. In 2016, alcoholic liver disease accounted for 15 148 of 25 213 alcohol-induced deaths (60.1%) among men and 6665 of 9644 (69.1%) among women. Deaths due to accidental poisoning by and exposure to alcohol or mental and behavioral disorders due to alcohol accounted for 9039 deaths (35.9%) among men and 2717 (28.2%) among women. The remaining 1026 deaths (4.1%) among men and 262 deaths (2.7%) among women were accounted for by a range of causes of alcohol-induced death.

The rate of death due to alcohol-induced causes overall increased from 2000 to 2016 at an AAPC of 1.4% (95% CI, 1.0%-1.8%) among men and 3.1% (95% CI, 2.6%-3.6%) among women. However, joinpoint analysis identified an accelerated increase in the most recent years; among men, the APC for 2012 to 2016 was 4.2% (95% CI, 3.1%-5.3%), while among women the APC from 2013 to 2016 was 7.1% (95% CI, 5.1%-9.1%) (Table).

**Table.** Age-Standardized Rates of Alcohol-Induced Deaths and APCs, 2000 to 2016

Racial/Ethnic Group	Men				Women			
	Period	No. of Deaths per 100 000 Residents^a^		APC (95% CI), %	Period	No. of Deaths per 100 000 Residents^a^		APC (95% CI), %
		Start	End			Start	End	
All	2000-2005	14.4	13.9	−0.6 (−1.5 to 0.3)	2000-2006	4.1	4.4	0.9 (0.1 to 1.8)
	2005-2012	13.9	15.3	1.3 (0.6 to 1.9)	2006-2013	4.4	5.4	3.4 (2.6 to 4.1)
	2012-2016	15.3	17.9	4.2 (3.1 to 5.3)	2013-2016	5.4	6.6	7.1 (5.1 to 9.1)
Non-Latino white	2000-2011	12.9	14.8	1.4 (1.0 to 1.7)	2000-2006	3.9	4.6	2.4 (1.5 to 3.2)
	2011-2016	14.8	18.2	4.4 (3.3 to 5.4)	2006-2013	4.6	6.0	4.1 (3.3 to 4.9)
					2013-2016	6.0	7.6	7.8 (5.7 to 9.9)
Latino/Latina	2000-2003	24.4	21.0	−5.1 (−9.8 to −0.1)	2000-2012	3.3	3.8	1.0 (0.1 to 1.9)
	2003-2013	21.0	19.6	−0.6 (−1.4 to 0.2)	2012-2016	3.8	4.7	5.6 (2.0 to 9.4)
	2013-2016	19.6	21.9	4.1 (0.3 to 8.1)				
Non-Latino black	2000-2006	19.6	13.0	−6.2 (−7.5 to −4.9)	2000-2007	5.6	3.5	−5.9 (−8.2 to −3.6)
	2006-2012	13.0	12.2	−1.7 (−3.6 to 0.2)	2007-2016	3.5	4.6	3.1 (1.5 to 4.8)
	2012-2016	12.2	13.8	2.7 (0.2 to 5.4)				
API	2000-2016	3.5	4.4	1.2 (0.3 to 2.1)	2000-2016	0.9	1.0	2.2 (0.5 to 4.0)
AIAN	2000-2016	66.9	113.2	3.3 (2.6 to 4.0)	2000-2016	30.7	58.8	4.2 (3.8 to 4.6)

Abbreviations: AIAN, American Indian and Alaska Native; APC, annual percentage change; API, Asian and Pacific Islander.

^a^ Rates are for single years at time interval boundaries.

### Difference Among Racial/Ethnic Groups

In 2016, the highest age-standardized rate was observed among the AIAN population (113.2 and 58.8 deaths per 100 000 residents among men and women, respectively) (Table). The next highest rate of death among men occurred among Latino men (21.9 deaths per 100 000 residents) followed by white men (18.2 deaths per 100 000 residents), black men (13.8 deaths per 100 000 residents), and API men (4.4 deaths per 100 000 residents). After AIAN women, the next highest rates of death occurred among white women (7.6 deaths per 100 000 residents), followed by Latina women (4.7 deaths per 100 000 residents), black women (4.6 deaths per 100 000 residents), and API women (1.0 deaths per 100 000 residents).

From 2000 to 2016, there was a significant increase in the rate of alcohol-induced deaths among AIAN men (AAPC, 3.3%; 95% CI, 2.6% to 4.0%), white men (AAPC, 2.3%; 95% CI, 2.0% to 2.7%), and API men (AAPC, 1.2%; 95% CI, 0.3% to 2.1%). In contrast, a significant decrease occurred among black men (AAPC, −2.4%; 95% CI, −3.3% to −1.4%), and no significant change occurred among Latino men. Among women, there were significant increases in all groups except black women, and these increases occurred more rapidly than among men (AIAN: AAPC, 4.2%; 95% CI, 3.8% to 4.6%; white: AAPC, 4.1%; 95% CI, 3.6% to 4.7%; API: AAPC, 2.2%; 95% CI, 0.5% to 4.0%; Latina: AAPC, 2.1%; 95% CI, 1.1% to 3.1%).

With the exception of AIAN and API men and women, among whom we observed steady increases in alcohol-induced deaths over the study period, trends were not consistent among other racial/ethnic groups (Table and Figure 1). For black women, the overall decrease from 2000 to 2016 reflected both a large decrease between 2000 and 2007 (APC, −5.9%; 95% CI, −8.2% to −3.6%) and a subsequent increase from 2007 to 2016 (APC, 3.1%; 95% CI, 1.5% to 4.8%). Similarly, whereas the overall trend among black men decreased from 2000 to 2016, the rate decreased substantially between 2000 and 2006 (APC, −6.2%; 95% CI, −7.5% to −4.9%), slowed between 2006 and 2012 (APC, −1.7%; 95% CI, −3.6% to 0.2%), and then increased from 2012 to 2016 (APC, 2.7%; 95% CI, 0.2% to 5.4%).

Among white men and women, there was a general increase from 2000 to 2016, although the largest increases occurred in the most recent years, particularly among women (2013-2016: APC, 7.8%; 95% CI, 5.7% to 9.9%). Among Latino men, there was a decline from 2000 to 2013 (2000-2003: APC, −5.1%; 95% CI, −9.8% to −0.1%; 2003-2013: APC, −0.6%; 95% CI, −1.4% to 0.2%) but a later increase from 2013 to 2016 (APC, 4.1%; 95% CI, 0.3% to 8.1%). For Latina women, there was an observed increase from 2000 to 2016, with the largest increase occurring from 2012 to 2016 (APC, 5.6%; 95% CI, 2.0% to 9.4%).

### Age Differences

**Figure 2.**
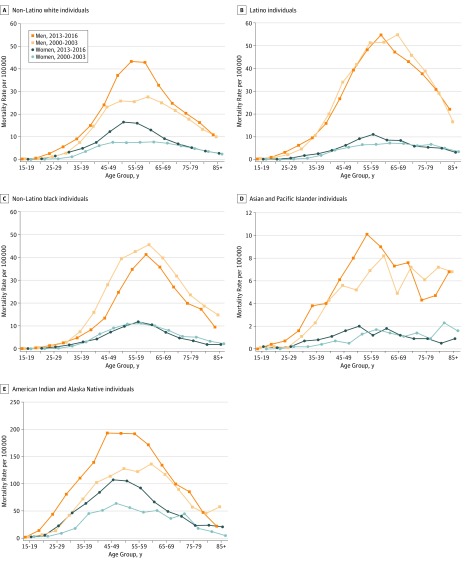
Comparison of Absolute Mortality Rates of Alcohol-Induced Deaths in 2013 to 2016 With Rates in 2000 to 2003

Considering the most recent period, ie, 2013 to 2016, peak mortality occurred between the ages of 55 and 64 years for all groups except the AIAN group (non-Latino white: men, 43.3 deaths per 100 000 residents; women, 16.5 deaths per 100 000 residents; Latino: men, 54.7 deaths per 100 000 residents; women, 11.1 deaths per 100 000 residents; non-Latino black: men, 41.2 deaths per 100 000 residents; women, 11.8 deaths per 100 000 residents; API: men, 10.1 deaths per 100 000 residents; women, 2.0 deaths per 100 000 residents) (Figure 2). Among AIAN individuals, peak mortality occurred between the ages of 45 and 49 years (men, 193.1 deaths per 100 000 residents; women, 107.1 deaths per 100 000 residents).

**Figure 3.**
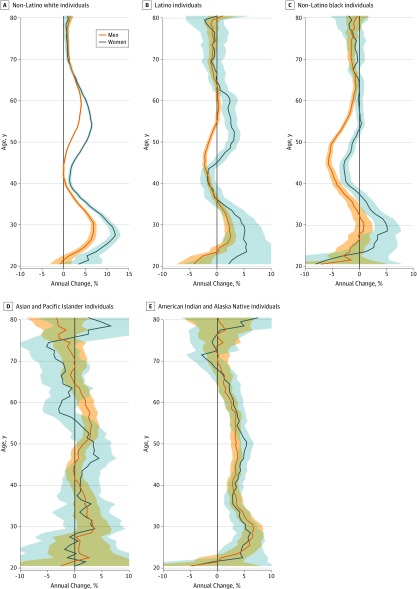
Estimated Annual Percentage Change in Rates of Alcohol-Induced Deaths From 2000 to 2016 by Age Shaded areas indicate 95% CIs.

Among AIAN men and women, large absolute increases occurred throughout the age range, with the largest absolute increase occurring for ages 45 to 49 years among men (2000-2013: 113.6 deaths per 100 000 residents; 2013-2016: 193.1 deaths per 100 000 residents) and for ages 50 to 54 among women (2000-2013: from 56.1 deaths per 100 000 residents; 2013-2016: 105.1 deaths per 100 000 residents) (Figure 2); APCs were generally similar by age (Figure 3). For example, increases from ages 23 to 60 years ranged from 2.4% per year to 6.7% per year among men and from 2.8% per year to 6.1% per year among women, with no clear pattern. Among white individuals, increases in absolute rates from 2000 to 2003 and from 2013 to 2016 also occurred through much of the age range but particularly in midlife (eg, men aged 55-59 years, 2000-2003: 25.5 deaths per 100 000 residents; 2013-2016: 43.3 deaths per 100 000 residents; women aged 50-54 years, 2000-2003: 7.4 deaths per 100 000 residents; 2013-2016: 16.5 deaths per 100 000 residents) (Figure 2); APCs for ages 25 to 34 years ranged from 4.6% per year to 6.9% per year among white men and from 7.3% to 12.0% per year among white women by age (Figure 3). Among black men, decreases occurred broadly throughout the age range and were largest between the ages of 38 and 54 years, with APCs ranging from −3.1% per year to −5.7% per year. Decreases at most ages were more modest among black women, and rates increased between the ages of 24 and 36 years, with APCs ranging from 1.3% per year to 5.1% per year (Figure 2 and Figure 3). Absolute increases among Latina women were largest between the ages of 45 and 69 years (ie, increases ranging from 0.9 deaths per 100 000 residents to 4.4 deaths per 100 000 residents across the age groups in this range) (Figure 2), although APCs were highest for Latina women aged approximately 30 years, ranging from 2.7% per year to 5.4% per year (Figure 3). Among API individuals, statistically significant APCs were observed among men aged 29 to 32 years (3.0%-3.5% per year) and aged 50 to 60 years (1.9%-3.1% per year) and among women aged 46 to 51 years (3.4%-4.4% per year) (Figure 3).

To further explore the recent increases in mortality among black and Latino individuals, we compared age-specific rates in 2013 to 2016 with those in 2009 to 2012. We found that the largest absolute increases occurred between the ages of 60 and 64 years among Latino men (2009-2012: 45.8 deaths per 100 000 residents; 2013-2016: 54.7 deaths per 100 000 residents), between the ages of 65 and 69 years among black men (2009-2012: 31.4 deaths per 100 000 residents; 2013-2016: 35.7 deaths per 100 000 residents) and between the ages of 55 and 59 years among black women (2009-2012: 8.4 deaths per 100 000 residents; 2013-2016: 11.8 deaths per 100 000 residents) (eFigure 2 in the Supplement).

In addition to age associations, we also observed higher rates of alcohol-induced deaths among more recent birth cohorts of non-Latino white and AIAN men and women (eFigure 3 in the Supplement). Relative to the reference cohort (ie, those born in 1958), the cohort rate ratio increased among white men to 2.76 (95% CI, 2.41-3.16) for the cohort born in 1986, while the cohort rate ratio among white women was 6.92 (95% CI, 5.34-8.98) for the cohort born in 1989. Among AIAN men, the corresponding rate ratio was 4.97 (95% CI, 3.49-7.09) for the cohort born in 1986, while among AIAN women the rate ratio was 5.29 (95% CI, 3.06-9.13) for those born in 1988. Increases also tended to be larger in the most recent periods (eFigure 4 in the Supplement). Relative to 2008 (ie, the midpoint of our study), the rate ratio among men progressively increased from 1.03 (2010 vs 2008) to 1.31 (2016 vs 2008), while the corresponding rate ratios among women increased from 1.10 to 1.58.

### Trends in Alcohol-Induced Deaths by State and County

**Figure 4.**
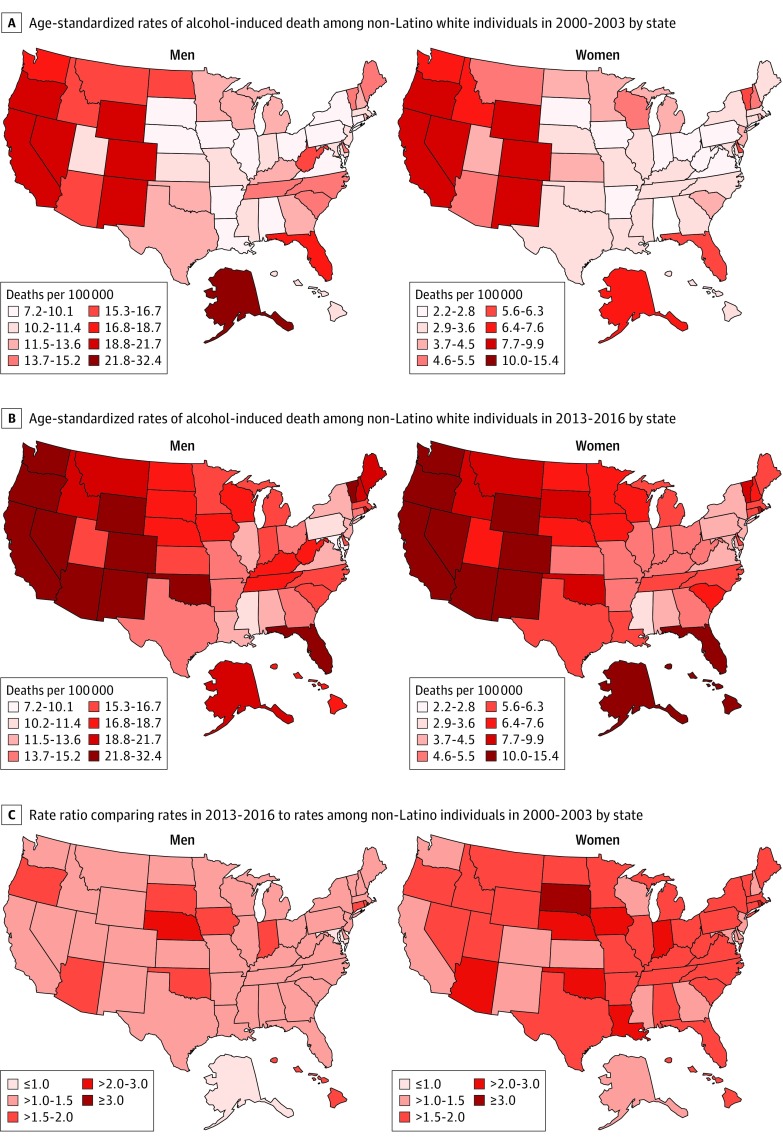
Age-Standardized Rates of Alcohol-Induced Death for Non-Latino White Individuals by State, Comparing 2000-2003 With 2013-2016

Among white individuals, rates of alcohol-induced deaths tended to be higher in states in the western United States, such as Oregon (2013-2016: men, 32.4 deaths per 100 000 residents; women, 12.9 deaths per 100 000 residents) and Wyoming (2013-2016: men, 28.8 deaths per 100 000 residents; women, 13.9 deaths per 100 000 residents). However, increases occurred in states throughout the country between 2000 to 2003 and 2013 to 2016 (eg, Iowa: men, rate ratio, 1.87; women, rate ratio, 2.37; Rhode Island: men, rate ratio, 1.56; women, rate ratio, 2.46) (Figure 4). Increases among white men and women occurred throughout urban, rural, wealthier, and poorer counties, as indicated by APCs greater than 1% (eFigure 5 in the Supplement).

## Discussion

The rate of alcohol-induced deaths, largely due to alcoholic liver disease, increased substantially among men and women in the United States from 2000 to 2016, especially in more recent years. Rates increased throughout the study period among AIAN men and women, white men and women, API men and women, and Latina women. Although declines occurred among black women from 2000 to 2007, black men from 2000 to 2012, and Latino men from 2000 to 2013, these promising trends reversed course, and rates of alcohol-induced mortality increased from 2013 to 2016 in all examined racial/ethnic groups.

In keeping with previous US reports highlighting morbidity and mortality among white individuals at midlife,^2^ we observed large absolute increases in alcohol-induced deaths within this group, particularly among men. However, the steepest increases in the rates of alcohol-induced deaths among white individuals in our study population occurred among younger adults, particularly women.

Our results for mortality are generally supported by prior studies identifying increases in alcohol use, high-risk drinking, and alcohol use disorders over recent years, particularly among women and other population subgroups not traditionally recognized as being at high risk.^6,18,19,20,21^ While we noted widespread increases geographically, our observation that the highest rates of death among white individuals occurred in the western United States echoes prior observations that states within this region had some of the highest historical per capita alcohol consumption levels.^21^ However, alcohol consumption levels are unlikely to fully explain mortality trends. Lack of access to high quality care for alcohol misuse and alcohol-associated diseases plays an important role in mortality vs morbidity.^21^ Indeed, alcohol-induced deaths should be considered a function of both alcohol misuse and insufficient primary, secondary, and tertiary prevention.

In our study, the largest absolute burden of alcohol-induced death and the largest absolute increases occurred among AIAN individuals. National surveys indicate that AIAN individuals are less likely to drink alcohol than other groups, but those who consume alcohol are more likely to drink larger amounts and to binge drink.^22,23^ Within the AIAN population, alcohol misuse should be considered within the context of historical trauma and exposure to other risk factors, which include poverty, family history of alcohol use disorder, availability of alcohol at a younger age through peer groupings that include older relatives, and acculturation stress.^22^ Additionally, available treatment and testing interventions have largely been developed for other populations and are poorly suited to AIAN populations.^21^ Dramatic underfunding of the Indian Health Service and underallocation of funding for mental health and substance use disorder services are also associated with alcohol-related morbidity and mortality.^24^

Unlike rates of alcohol-induced death among other groups, which steadily increased from 2000 to 2016, rates among black men and women and Latino men declined over much of the period before increasing in later years. Our results for initial declines have been observed previously.^1,25^ Explanations for these declines and subsequent increases remain to be determined and speak to the need for studies that carefully investigate trends in both alcohol use and access to appropriate health care and treatment by race/ethnicity, sex, and birth cohort.^25^

Although the current study focuses on the United States, it is important to acknowledge that alcohol is a leading cause of premature mortality worldwide.^26^ The highest levels of alcohol consumption have consistently occurred in Europe, despite efforts by European Union member states to make alcohol consumption a public health priority, indicating the substantial challenge of alcohol-induced disease.^27^ Rates of alcohol-induced deaths in the United States have now reached those of the United Kingdom, where the rate of alcohol-specific deaths in 2016 was 11.7 deaths per 100 000 residents.^28^ However, rates in the United Kingdom have been largely stable since 2013, in contrast with the rapid increases we observed during this period in the United States. In Canada, increases from 2001 to 2017 have also been noted, particularly among women.^29^ Clearly, alcohol-induced deaths are a major problem worldwide, meriting a substantial public health response.^26^

The US Preventive Services Task Force has recently recommended screening for unhealthy alcohol use among adults in primary care settings as well as the provision of behavioral counseling interventions.^30^ Similarly, the American Society of Clinical Oncology recently published a statement on alcohol and cancer providing guidance for reducing the disease burden.^31^ Stronger alcohol regulations have been associated with lower alcohol consumption, less binge drinking, and lower rates of mortality from alcoholic cirrhosis.^32^ However, in the case of both screening^22^ and regulation,^32^ appropriateness and utility of interventions may vary with racial/ethnic and regional differences, in accordance with underlying risk factors, cultural factors, and health care provision disparities, demanding the deployment of sophisticated and culturally aware interventions.^33^

Our study benefits from a thorough consideration of trends in alcohol-induced death rates across individual population subgroups using the full US population older than 15 years. Previous studies have described US mortality trends but focused on certain causes. For example, in 2018, Tapper and Parikh^34^ described mortality due to cirrhosis and liver cancer, and several studies have examined the association of alcoholic liver disease and alcohol-induced deaths in rising rates of premature mortality in the United States.^2,3,5^ We included all causes of death known definitively to be induced by alcohol, providing an unambiguous measure of deaths of which alcohol is the sole cause.^35^ Furthermore, we have examined how trends differed by sex, race/ethnicity, age, county-level SES, and geographic location. To improve accessibility, we have predominantly presented our results graphically, including displaying uncertainty surrounding our joinpoint estimates.

### Limitations

This study has limitations. Because we restricted our outcome definition to alcohol-induced deaths, we excluded causes known to be associated with alcohol but not 100% attributable to alcohol (eg, traffic collisions, alcohol-associated cancers, infections and organ system diseases known to be associated with alcohol use). Thus, although our analysis of trends in alcohol-induced deaths provides an important indicator of the consequences of alcohol on population health, our findings substantially underestimate the full mortality burden.^26,36^ Our study also bears limitations associated with the underreporting of alcohol-attributable deaths on death certificates.^37^ Polednak^37^ observed that use of multiple-cause death records, as opposed to the underlying cause of death, may enhance surveillance of premature mortality because of chronic (although not acute) disease resulting from alcohol use; we encourage future research that considers the effects of this approach. Conversely, we recognize that deaths classified as alcohol-induced deaths may have been influenced by coexisting conditions otherwise affecting the liver (eg, hepatitis C infection or nonalcoholic fatty liver disease).^38,39^ Misclassification of race/ethnicity data within death certificates is also possible in our study but is minimal for groups other than AIAN individuals, for whom we took measures to limit misclassification.^3^ Also, our analyses of county-level factors were limited to white, black, and Latino individuals, and our analysis by state was limited to white individuals; these exclusions were made because of sparse data for minority groups within the subdivisions.

## Conclusions

This study showed increases in the rate of alcohol-induced deaths across racial/ethnic subgroups of the US population, which have accelerated during recent years. Because many of the consequences of alcohol consumption occur later in life, large increases in alcohol-induced deaths among younger age groups portend substantial future increases in alcohol-related disease. Thus, narratives regarding increasing deaths among white US residents at midlife should be extended to note large increases in rates of alcohol-induced death among women and younger groups as well as among minority populations. Reflecting on the consequences of alcohol-related morbidity and mortality throughout the age range, our findings document an urgent public health crisis calling for concerted public health action.

## Notes

Supplement. **eFigure 1. **Age-Standardized Rates of Alcohol-Induced Deaths, 2000-2016, by Sex and Specific Cause **eFigure 2. **Male and Female Age-Specific Rates of Alcohol-Induced Deaths for 2013-2016 vs 2009-2012 for Latino/Latina and non-Latino Black Individuals **eFigure 3. **Male and Female Birth Cohort Effects Derived From Age-Period-Cohort Models for Persons Aged 20-80 Years, Inclusive **eFigure 4. **Male and Female Time Period Effects Derived From Age-Period-Cohort Models for Persons Aged 20-80 Years, Inclusive **eFigure 5. **Annual Percentage Change in Alcohol-Induced Mortality Rate, 2000-2016, by County-Level Socioeconomic Status (SES) and Rurality
