# Supplementary material for: Trends in Alcohol-Induced Deaths in the United States, 2000-2016
Source: JAMA Netw Open. 2020 Feb 21;3(2):e1921451. doi: 10.1001/jamanetworkopen.2019.21451 (PMC7043198; doi:10.1001/jamanetworkopen.2019.21451)
Supplement: Supplement. — eFigure 1. Age-Standardized Rates of Alcohol-Induced Deaths, 2000-2016, by Sex and Specific Cause eFigure 2. Male and Female Age-Specific Rates of Alcohol-Induced Deaths for 2013-2016 vs 2009-2012 for Latino/Latina and non-Latino Black Individuals eFigure 3. Male and Female Birth Cohort Effects Derived From Age-Period-Cohort Models for Persons Aged 20-80 Years, Inclusive eFigure 4. Male and Female Time Period Effects Derived From Age-Period-Cohort Models for Persons Aged 20-80 Years, Inclusive eFigure 5. Annual Percentage Change in Alcohol-Induced Mortality Rate, 2000-2016, by County-Level Socioeconomic Status (SES) and Rurality [file jamanetwopen-3-e1921451-s001.pdf]

Supplementary Online Content

Spillane S, Shiels MS, Best AF, et al. Trends in alcohol-induced deaths in the United States, 2000-2016. *JAMA Netw Open*. 2020;3(2):e1921451. doi:10.1001/jamanetworkopen.2019.21451

eFigure 1. Age-Standardized Rates of Alcohol-Induced Deaths, 2000-2016, by Sex and Specific Cause

eFigure 2. Male and Female Age-Specific Rates of Alcohol-Induced Deaths for 2013-2016 vs 2009-2012 for Latino/Latina and non-Latino Black Individuals

eFigure 3. Male and Female Birth Cohort Effects Derived From Age-Period-Cohort Models for Persons Aged 20-80 Years, Inclusive

eFigure 4. Male and Female Time Period Effects Derived From Age-Period-Cohort Models for Persons Aged 20-80 Years, Inclusive

eFigure 5. Annual Percentage Change in Alcohol-Induced Mortality Rate, 2000-2016, by County-Level Socioeconomic Status (SES) and Rurality

This supplementary material has been provided by the authors to give readers additional information about their work.

© 2020 Spillane S et al. *JAMA Network Open.*

eFigure 1. Age-Standardized Rates of Alcohol-Induced Deaths, 2000-2016, by Sex and Specific Cause

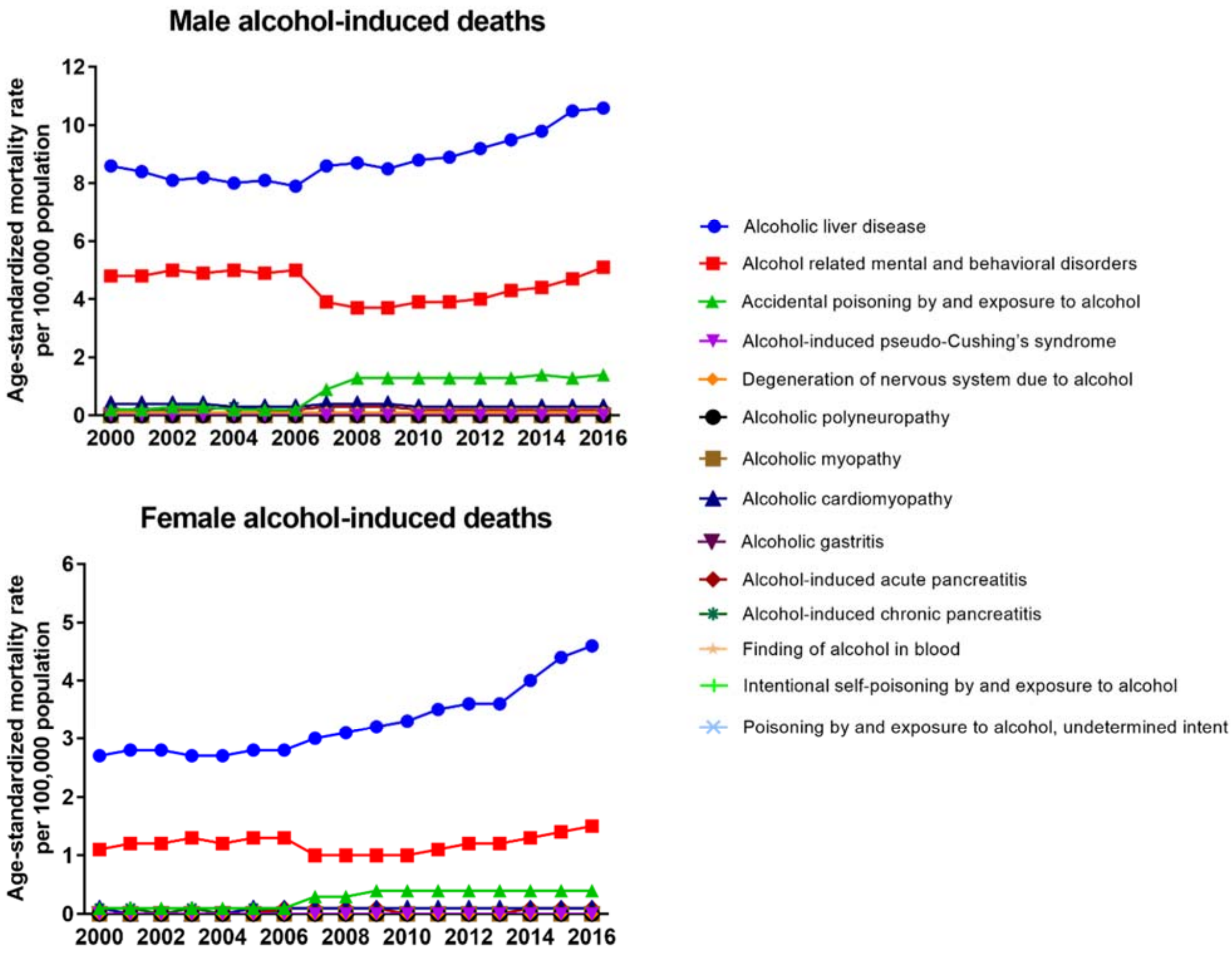

© 2020 Spillane S et al. *JAMA Network Open.*

eFigure 2. Male and Female Age-Specific Rates of Alcohol-Induced Deaths for 2013-2016 vs 2009-2012 for Latino/Latina and non-Latino Black Individuals

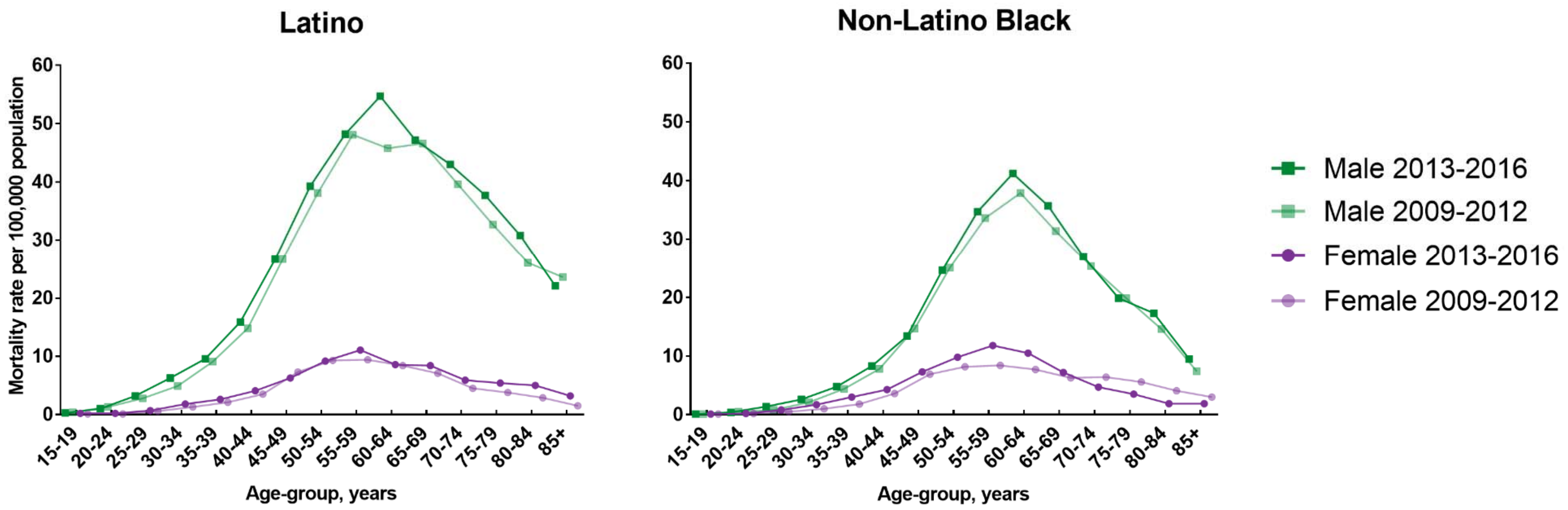

© 2020 Spillane S et al. *JAMA Network Open.*

eFigure 3. Male and Female Birth Cohort Effects Derived From Age-Period-Cohort Models for Persons Aged 20-80 Years, Inclusive

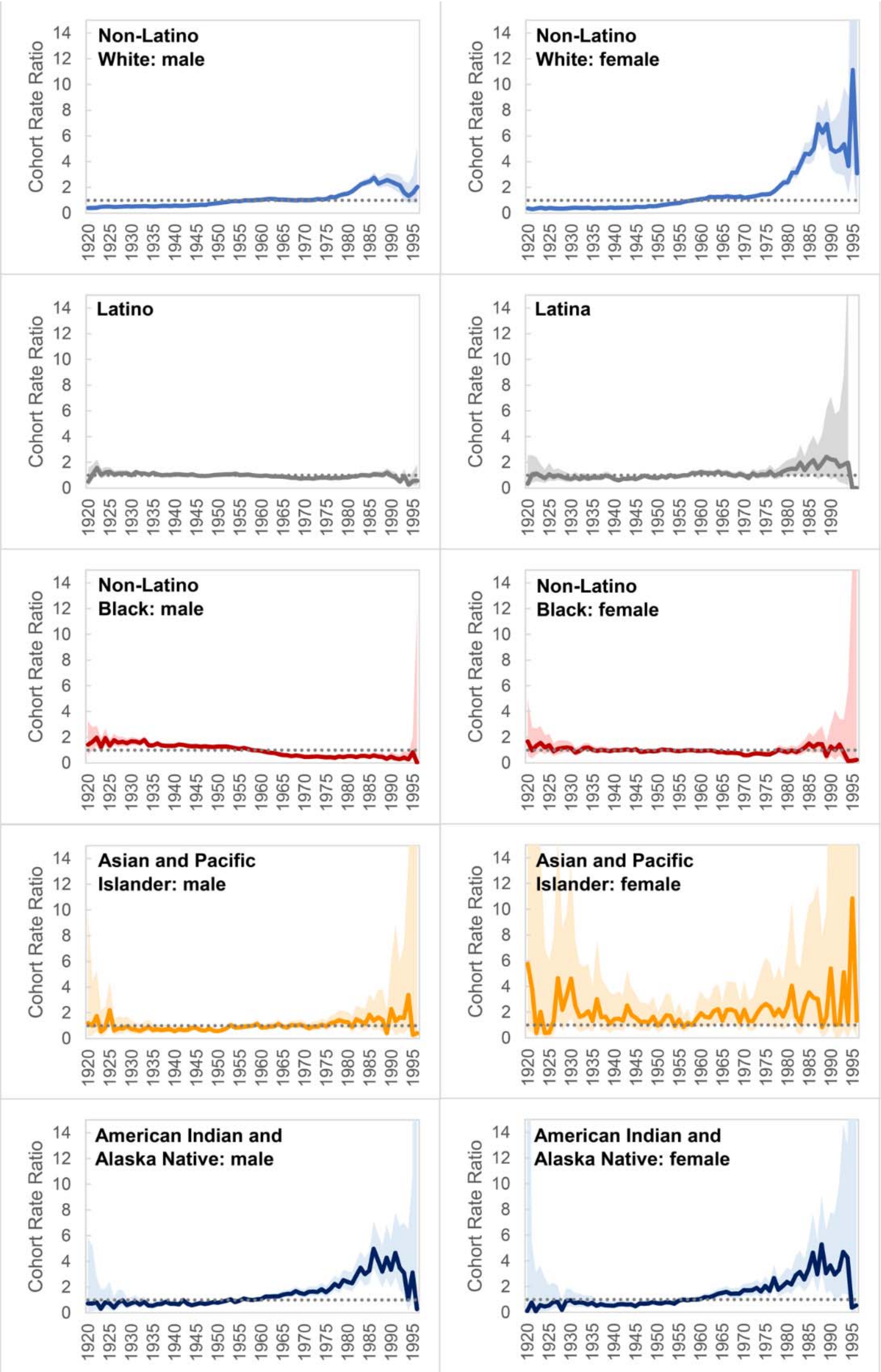

Alcohol-induced mortality rate ratios (with shaded 95% confidence intervals) for each birth cohort, 1920-1996, are expressed relative to the middle birth cohort (1958) (rate ratio =1, depicted with dotted line). Due to sparsity of data 1994-1996, Latina rate ratios are expressed relative to 1956. Y-axis maximum set to “15” due to extremely high upper confidence intervals in some groups for the most recent birth cohorts.

© 2020 Spillane S et al. *JAMA Network Open.*

eFigure 4. Male and Female Time Period Effects Derived From Age-Period-Cohort Models for Persons Aged 20-80 Years, Inclusive

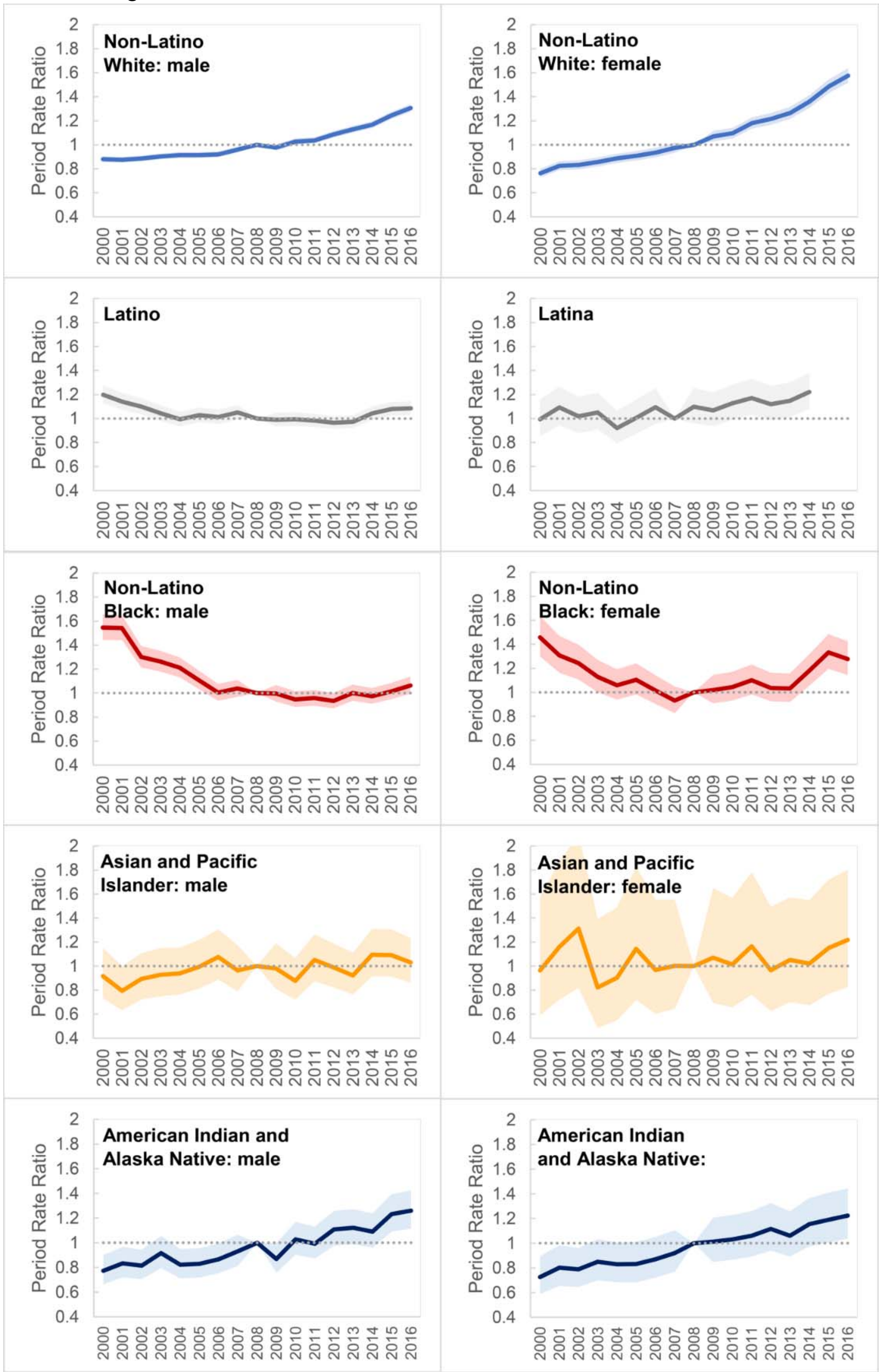

Alcohol-induced mortality rate ratios (with shaded 95% confidence intervals) for the midpoint of each year, 2000-2016, are expressed relative to the mid-point (2008) (Due to sparsity of data, Latina rate ratios are expressed relative to 2007).

© 2020 Spillane S et al. *JAMA Network Open.*

eFigure 5. Annual Percentage Change in Alcohol-Induced Mortality Rate, 2000-2016, by County-Level Socioeconomic Status (SES) and Rurality

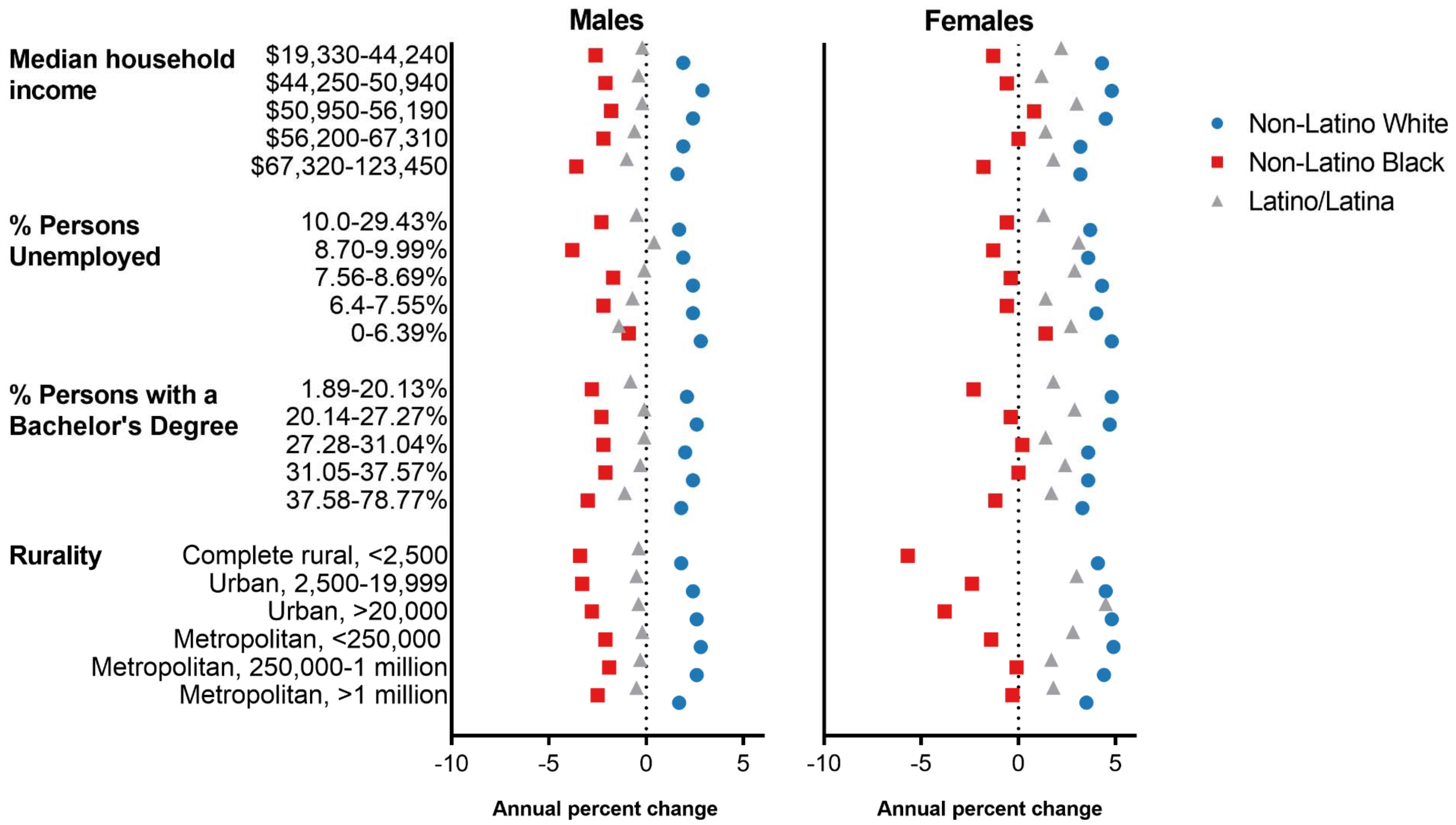

© 2020 Spillane S et al. *JAMA Network Open.*
